# Acupuncture point injection markedly improved sensory symptoms and motor signs in 2 patients with restless legs syndrome

**DOI:** 10.1002/ccr3.1619

**Published:** 2018-05-31

**Authors:** Takero Fukutome

**Affiliations:** ^1^ Fukuoka Sleep Clinic Fukuoka Japan

**Keywords:** acuinjection, acupuncture, polysomnography, restless legs syndrome

## Abstract

The acuinjections at acupuncture points (GB41, BL60, ST36, and SP6) provide immediate relief of sensory symptoms and motor signs of restless legs symptom (RLS). An acuinjection can be promising and safe alternative treatment for pharmacotherapy in patients of RLS.

## 1 INTRODUCTION

Two patients with restless legs syndrome (RLS) who complained of uncomfortable leg sensations and nonrestorative sleep underwent acupuncture injection treatment at acupuncture points (GB41, BL60, ST36, and SP6) in both legs during polysomnography. Immediately afterward, their complaints and periodic leg movements were relieved resulting in restorative sleep.

Restless legs syndrome (RLS), or Willis‐Ekbom disease, is a sensorimotor disorder that frequently involves periodic limb movements during sleep and is a major sleep‐related disorder. It is characterized by an uncomfortable sensation in the limbs and an irresistible urge to move them.^1^

There have been few English‐language reports of acupuncture for RLS. According to a Cochrane review, 2 randomized controlled trials have been reported in Chinese‐language papers. Although one paper reported no significant clinical effects, the other (n = 90, relative risk = 1.36) reported remission of unpleasant leg sensations, but this paper had methodological problems including improper randomization. The effects are thus not conclusively established.^2^ In a recent review,^3^ it was concluded for acupuncture that “there is no sufficient evidence from well‐conducted randomized trials to recommend its use in RLS.” In another recent review,^4^ quoting a report by Pan et al,^5^ the authors speculated that acupuncture is a promising treatment method, but its clinical utility was not addressed.

Detailed assessment of the clinical reports of acupuncture treatment for RLS is important. Three major reports written in English were located on PubMed and Google Scholar.

In the first report,^6^ acupuncture in the several acupoints combined with acupuncture injections (acuinjections) using *Radix Salviae Miltiorrhiza*e was performed on 49 patients with RLS, and after 6 months of observation, complete symptom relief was achieved in 41 patients. However, the selection method among ten acupoints (BL 18. 23. 56. 57. 64, SP 5. 6. 10, GB 34, KI 3) was dependent on Traditional Chinese Medicine (TCM), and the needling manipulation followed the classic acupuncture technique. Both the selection method and the classic needling technique may be challenging for Western physicians. Moreover, the diagnostic criteria of RLS are not clarified and the pharmacological safety of *Radix Salviae Miltiorrhizae* has not been established.

The second report^7^ examined the differences of electroacupuncture's effects at 2 acupoints (BL 57 and LR 3) among patients with RLS who either had or had not been previously treated with dopaminergic drugs. The study followed a retrospective, survey design with a patient memory‐dependent questionnaire to evaluate symptoms after 6 months of treatment. In the patients that had not been previously treated with dopaminergic drugs, electroacupuncture relieved symptoms from 8.9 to 3.1 on the visual analog scale (with 10 being the worst possible score and 0 the best); the effect was greater than that in patients who had been previously treated with dopaminergic drugs. However, electroacupuncture requires a dedicated device, and detailed clinical observation of the effects has not been reported.

In the third report,^5^ in 38 patients with RLS, the differences of activity on the actigram among acupuncture‐treated and sham‐treated patients were observed for 6 months. Actigram's nocturnal activity decreased significantly in acupuncture‐treated patients compared with sham‐treated patients (30.1 vs 10.0), and the International RLS Study Group rating scale (IRLS)^8^ score decreased (5.7 vs 1.3). However, acupuncture was performed at 12 acuspots (BL23. 57, SP6. 10, LR3, St36, KI3, GV4: unilateral or bilateral) simultaneously, using the classic acupuncture manipulation technique, by an experienced acupuncturist, 3 times per week for 6 weeks. As a result, 4 patients (10.5%) dropped out due to pain related to acupuncture. Such excessively invasive acupuncture is difficult to use clinically.

Thus, there is no report supporting a clinically simple and effective acupuncture treatment for RLS. Here, I report 2 cases of RLS that remarkably improved with simplified acuinjections. The effects were confirmed by polysomnography (PSG) in addition to subjective improvement.

## 2 METHODS

Acupuncture is part of TCM, which may be challenging for Western physicians to learn. Modified acupuncture methods, including acuinjection and electroacupuncture, were developed resulting in a simplified technique. Acuinjection is an acupoint‐stimulating technique involving liquid agent injections instead of needle‐based acupoint manipulation. Although acuinjection requires no special technique, in contrast to classic acupuncture, no standardization in the selection of acuspots or dose and the solutions of the injection have been reported.

Simplified acupuncture was performed using the method developed by Watanabe^9^ (W‐acuinjection). In W‐acuinjection, a small normal saline dose is injected at an acupoint along the meridians leading to the affected area. When pentazocine or pethidine solutions are used instead of normal saline, stronger and sustained effects can be expected. Thus, in this method, the selection of acupoints and injections to be used is simplified, and it may be easily utilized by physicians unfamiliar with classic acupuncture.

**Figure 1:**
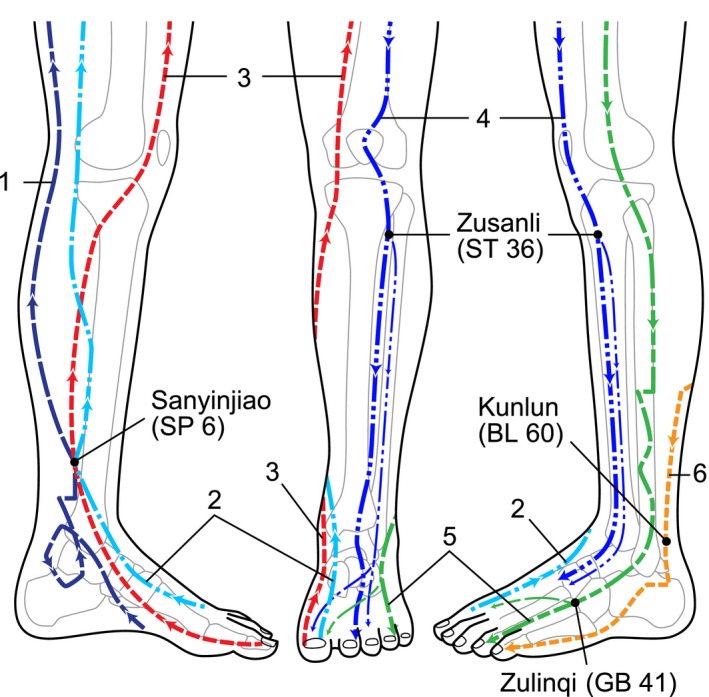
Acupuncture points and related meridians. Acupuncture points described according to World Health Organization standards^10^ Chinese phonetic alphabet name (alphanumeric code). Meridians: (1) kidney, (2) liver, (3) spleen, (4) stomach, (5) gallbladder, (6) bladder. This figure is adapted from an earlier work (9) with the author's kind permission

In this report, W‐acuinjections were administered to both legs. Normal saline doses (0.25 mL each) supplemented with pentazocine (0.5‐1 mg per dose) were acuinjected at the *Zulinqi* (GB41), *Kunlun* (BL60), *Zusanli* (ST36), and *Sanyinjiao* (SP6)^10^ (Figure 1) acupoints in both legs (4W‐acuinjection).

At each acupoint, a ¾‐inch‐long, 27‐gauge needle was inserted perpendicularly in the skin, and the dose was acuinjected at a depth of approximately 0.5 cm, except for the GB41 acupoint, where the needle was inserted just beneath the subcutaneous layer.

Attended PSG was performed in accordance with the American Academy of Sleep Medicine's guidelines^11^ with an Alice 6 LDxS (Philips Respironics, Murrysville, PA) or Embletta MPR PG (Natus Medical, Pleasanton, CA) device. An authorized physician of the Japanese Sleep Society or an authorized sleep technician collected the data and analyzed them with the Sleepware G3 (Philips Respironics) or Embla RemLogic PSG Software (Natus Medical).

Written informed consent was obtained from the 2 patients. The study was approved by the ethics committee of Fukuoka Sleep Clinic as complied with the Declaration of Helsinki.

## 3 REPORT OF CASES

### 3.1 Case 1

A 73‐year‐old man (body mass index: 23.5 kg/m^2^) complained of an urge to move the legs accompanied by an unpleasant sensation that he could not describe in words, worsened during rest and precipitated before night sleep, resulting in difficulty in falling asleep and nonrestorative sleep. His symptoms were relieved by mobilization and met the RLS diagnostic criteria.^12^ His IRLS score was 11. He felt very uncomfortable upon waking in the morning and claimed that even prolonged sleep did not relieve his fatigue. His average sleep duration was 10 hours. His physical examination and laboratory tests yielded normal findings, including a serous ferritin level of 69 ng/mL. He had been previously treated for hyperlipidemia and ischemic heart disease, and he declined the use of additional medication, apart from hypnotics.

**Figure 2:**
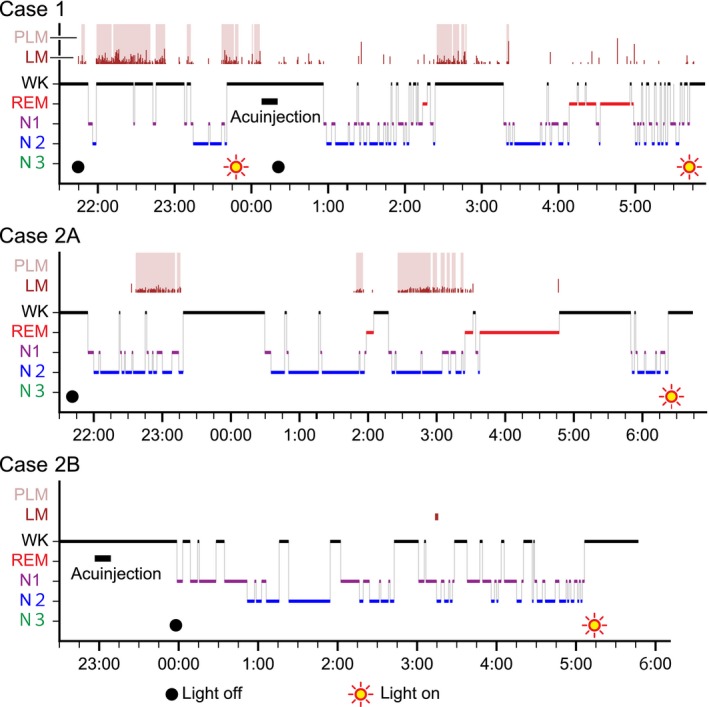
LMs/PLMs and somnograms before and after the acupoint injections. LM, leg movement; N1, Stage N1; N2, Stage N2; N3, Stage N3; PLM, periodic leg movement; REM, Stage R; WK, Stage W

I obtained a full‐night diagnostic PSG recording while the patient was on hypnotic medication (zolpidem, 5 mg). I found that he experienced difficulty in initiating and maintaining sleep due to the discomfort associated with periodic leg movement (PLM) bursts. He received 4W‐acuinjections of normal saline doses (0.25 mL each) supplemented with pentazocine (0.5 mg per dose) in both legs after the first 115 min of PSG. Immediately afterward, the patient felt a comfortable warmth to both legs, his discomfort subsided completely, and the PLMs ceased (Figure 2).

Although short PLM bursts reappeared 124 minutes later, the patient reported remarkably restorative sleep in the morning. Most of the PLMs were observed during stage wakefulness periods, where slow eye movements were observed while EEG was alpha‐rhythm dominant. Thus, discomfort accompanied by PLMs during drowsy periods, rather than wakefulness, seemed to disrupt his attempts to fall asleep.

After the 4W‐acuinjections, his PLM index (number of PLMs/total recording h) decreased, and his percent sleep efficiency (total sleep time/total recording time × 100%) increased (Table 1).

**Table 1:** Polysomnographic variables before and after the acupoint injections

	TRT (min)	AHI (h^−1^)	PLMI (h^−1^)	PLMSI (h^−1^)	ArI (h^−1^)	%SE (%)
Case 1						
Before acuinjection	125.3	1.7	84.5	10.4	29.6	32.1
After acuinjection	321.7	7.9	12.2	1.1	49.5	74.8
Case 2						
Before acuinjection	524.2	22.2^a^	21.8	32.2	43.2	69.7
After acuinjection	315	0.3^a^	0	0	26.7	75.1

%SE, percent sleep efficiency (sleep time as percentage of recording time); AHI, apnea‐hypopnea index (number of apneas and hypopneas per h of sleep); ArI, arousal index (number of electroencephalogram arousals per recording h); PLMI, periodic leg movement index (number of periodic leg movements per recording h); PLMS, number of periodic leg movements of sleep; PLMSI, PLMS index (number of periodic leg movements of sleep per sleep h); TRT, total recording time.

a AHI in supine position.

### 3.2 Case 2

An 81‐year‐old man experienced symptoms including interrupted sleep (ie, waking up every 2 hours), excessive daytime sleepiness, and intolerable fatigue throughout the day. He experienced unpleasant paresthesia of the left leg urging to move. The symptoms worsened at rest and precipitated during the evening and night and were relieved by mobilization. His symptoms met the RLS diagnostic criteria, with an IRLS score of 16. However, he involuntarily tapped his right heel once every few seconds but did not report any discomfort related to this. Ordinarily, leg discomfort is bilateral in RLS. The patient's discomfort in the right leg could have been masked by his tapping; thus, his symptoms were consistent with RLS's typical presentation.

His average sleep duration was 9.5 hours. He was receiving pharmacotherapy for panic disorder, hypertension, hyperlipidemia, and dyspepsia. He refused additional pharmacotherapy. Laboratory tests returned normal findings, except for a ferritin level of 34.7 ng/mL and a hypoproteinemic serum protein level of 6.6 g/dL. His physical examination was unremarkable, except for emaciation (body mass index: 17.0 kg/m^2^).

Diagnostic PSG revealed a high baseline obstructive apnea‐hypopnea index in the supine position (Table 1), which was associated with remarkable respiratory efforts, and a PLM index of 32.2, which was associated with bilateral gross knee flexion accompanied by EEG arousals (PLM arousal index during sleep: 10.3). All his PLMs occurred during sleep. No tapping‐like movements were observed when he lay on the bed with extended knees, which suggested that knee flexion induced the movements.

At‐home continuous positive airway pressure (CPAP) treatment was initiated with an auto‐titrating device. During a month‐long period preceding the follow‐up visit, the patient's average apnea‐hypopnea index was 0.7, and his compliance was 100%, but he continued experiencing discomfort of the left leg, right foot tapping, and nonrestorative sleep.

The second diagnostic PSG under auto‐titrated CPAP was obtained 2 months after the first PSG. Before the PSG, he received 4W‐acuinjections of normal saline doses (0.25 mL each) supplemented with pentazocine (1 mg per dose) in both legs in the sitting position while tapping his right heel, and immediately afterward, he felt warmth to both legs with a pleasant sensation, and his left lower leg discomfort and involuntary right leg movements were completely relieved. During the PSG, his PLMs were almost completely suppressed except for one leg movement. The 4W‐acuinjections caused no major change in percent sleep efficiency, but the patient reported remarkably restorative sleep upon waking up in the morning.

### 3.3 Postacuinjection care

Only a doctor or a nurse can carry out acupuncture legally and patients can't perform acupuncture by himself. Therefore, pharmacotherapy was applied to the treatment after the PSG.

Both patients accepted treatment with oral gabapentin enacarbil (Case 1) or rotigotine transdermal patches (Case 2) after experiencing the remarkably restorative sleep. Both treatments provided marked relief from the RLS symptoms, including the involuntary leg movements. Oral iron supplementation was initiated in Case 2.

No significant 4W‐acuinjection‐related complications, except mild injection‐related pain, were observed in either patient.

## 4 DISCUSSION

RLS is primarily treated with palliative pharmacotherapy, except for iron supplementation when iron deficiency is identified. However, pharmacotherapy can often be ineffective or have adverse effects.^13^

In this report, both patients were relieved of uncomfortable lower leg sensations and involuntary leg movements, including PLMs during wakefulness and sleep, immediately after 4W‐acuinjections and reported remarkably restorative sleep in the morning. Given the prompt responses, any systemic effects of the acuinjected normal saline or pentazocine can be excluded. Rapid decrease in symptoms and PLMs after the 4W‐acuinjection could be caused by the placebo effect or by the circadian rhythm of RLS. However, the immediate sensation of comfortable warmth in the legs and disappearance of involuntary leg movements after the 4W‐acuinjection strongly excludes these assumptions.

Chronic pain, including low back pain and knee pain, can be treated with acupuncture, which modulates transient receptor potential cation channel vanilloid 1 activation in the periphery, microglial suppression in the cerebral cortex and spinal cord, and regulation of cytokines and other key inflammatory factors in the spinal cord.^14^

From a neurophysiological perspective, the mechanism of chronic pain is similar to that of RLS. RLS is hypothesized to be a complex sensorimotor network disorder involving cortical, subcortical, spinal, and peripheral nerve generators and resulting in enhanced excitability and/or decreased inhibition.^15^

The involvement of these overlapping networks suggests that acupuncture can serve as a new tool for clarifying the pathological aspects of RLS.

I used the GB41, BL60, ST36, and SP6 acupoints, the first three of which belong to the 3 meridians passing through the leg's lateral side. The 3 meridians passing through the leg's inner side cross at the fourth acupoint, the SP6. I thus expected remission of uncomfortable lower leg sensations and PLMs following acuinjections at these 4 acupoints. However, whether all 4 acupoints are therapeutically essential requires evaluation.

This study has some limitations. First, percent sleep efficiency may be a suboptimal marker for sleep quality improvements in this study, as it might have been influenced by factors other than the acuinjections. Second, the observation period was limited to the peri‐PSG period. The PLMs, which were suppressed following the 4W‐acuinjections, reappeared during PSG in 1 patient. Thus, the 4W‐acuinjection's major effect tends to dissipate within a day.

However, even short‐acting treatment may be useful in some clinical occasions, including positive airway pressure(PAP)‐titration under PSG like in this report in a patient with obstructive sleep apnea complicated with RLS in where excluding of interference of the RLS upon the PAP‐titration is desirable.

In clinical situations of RLS treatment, the effect of treatment needs to last at least full‐night or more. The effects of the acuinjection in this report show that stimulation of the 4 acuspots is useful for treating RLS and does not exclude the effectiveness of other stimulation methods. For example, an intradermal acupuncture can be expected to be a long‐lasting treatment method substitute acuinjection treatment.

Thus, despite some limitations, 4W‐acuinjection may be an effective new option or suggestions of new methods for treating RLS.

## CONFLICT OF INTEREST

None declared.

## AUTHORS' CONTRIBUTIONS

TF: conceived the study, acquired, analyzed and interpreted the data, wrote the manuscript and is accountable for all aspects of the work.
